# Effects of copper overload on mitochondrial parameters in GBM-1, U-87 MG, and C6 glioma cell lines

**DOI:** 10.1007/s10534-026-00863-1

**Published:** 2026-07-25

**Authors:** Giovanna Tassinari, Lorena Aparecida de Souza, Nikole Aguiar de Souza, Yasmin Radowitz Mendonça, Lara Stoeberl, Madson Silveira de Melo, Evelise Maria Nazari, Carla Inês Tasca, Cláudia Beatriz Nedel, Viviane Glaser

**Affiliations:** 1 Laboratório de Biologia Celular, Centro de Ciências Rurais, Coordenadoria Especial de Ciências Biológicas e Agronômicas, Universidade Federal de Santa Catarina, Campus de Curitibanos, Curitibanos, SC Brazil; 2 Laboratório de Biologia Celular de Gliomas, Centro de Ciências Biológicas, Departamento de Biologia Celular, Embriologia e Genética, Universidade Federal de Santa Catarina, Campus Trindade, Florianópolis, SC Brazil; 3 Laboratório de Reprodução e Desenvolvimento Animal, Centro de Ciências Biológicas, Departamento de Biologia Celular, Embriologia e Genética, Universidade Federal de Santa Catarina, Campus Trindade, Florianópolis, SC Brazil; 4 Laboratório de Neuroquímica 4, Centro de Ciências Biológicas, Departamento de Bioquímica, Universidade Federal de Santa Catarina, Campus Trindade, Florianópolis, SC Brazil

**Keywords:** Metals, Mitochondria, PGC-1α, PINK1, TFAM

## Abstract

Over the years, there has been growing interest in developing new therapies for glioblastoma, and copper compounds have emerged as promising therapeutic targets due to their antitumoral properties. In this context, this study aimed to investigate the effects of high copper levels on cell viability, mitochondrial physiology, PINK1 content, and the expression of PGC-1α and TFAM in distinct glioma cell lines, mainly considering the heterogeneity of tumoral cells. GBM-1, U-87 MG, and C6 cells CuSO_4_ exposure (0–1200 µM) was performed for 24 h, and cell viability was assessed using the MTT reduction and Neutral Red (NR) assays. NADH dehydrogenase, succinate dehydrogenase, cytochrome c oxidase activities, and the mitochondrial membrane potential (ΔΨm) were measured to analyze mitochondrial physiology. PINK1 was evaluated by immunofluorescence, while PGC-1α and TFAM expression were assessed by real-time RT-PCR in U-87 MG and C6 cells. Copper exposure reduced cell viability in the cell lines (MTT and NR), except in U-87 MG with the NR assay. High copper levels decreased succinate dehydrogenase activity in GBM-1, U-87 MG, and C6 cells. Cytochrome c oxidase activity was decreased in the C6 cell line. Moreover, reductions in ΔΨm and increases in PINK1 immunostaining were observed across the three cell lines. Finally, an increase in PGC-1α mRNA was observed in C6 cells, and TFAM expression has risen in U-87 MG. In conclusion, high copper levels decrease glioma cell viability in vitro, and mitochondrial dysfunction contributes to this effect, resulting in PINK1 and TFAM or PGC-1α increase as compensatory mechanisms to copper cytotoxicity.

**Graphical abstract:**

**Figure:**
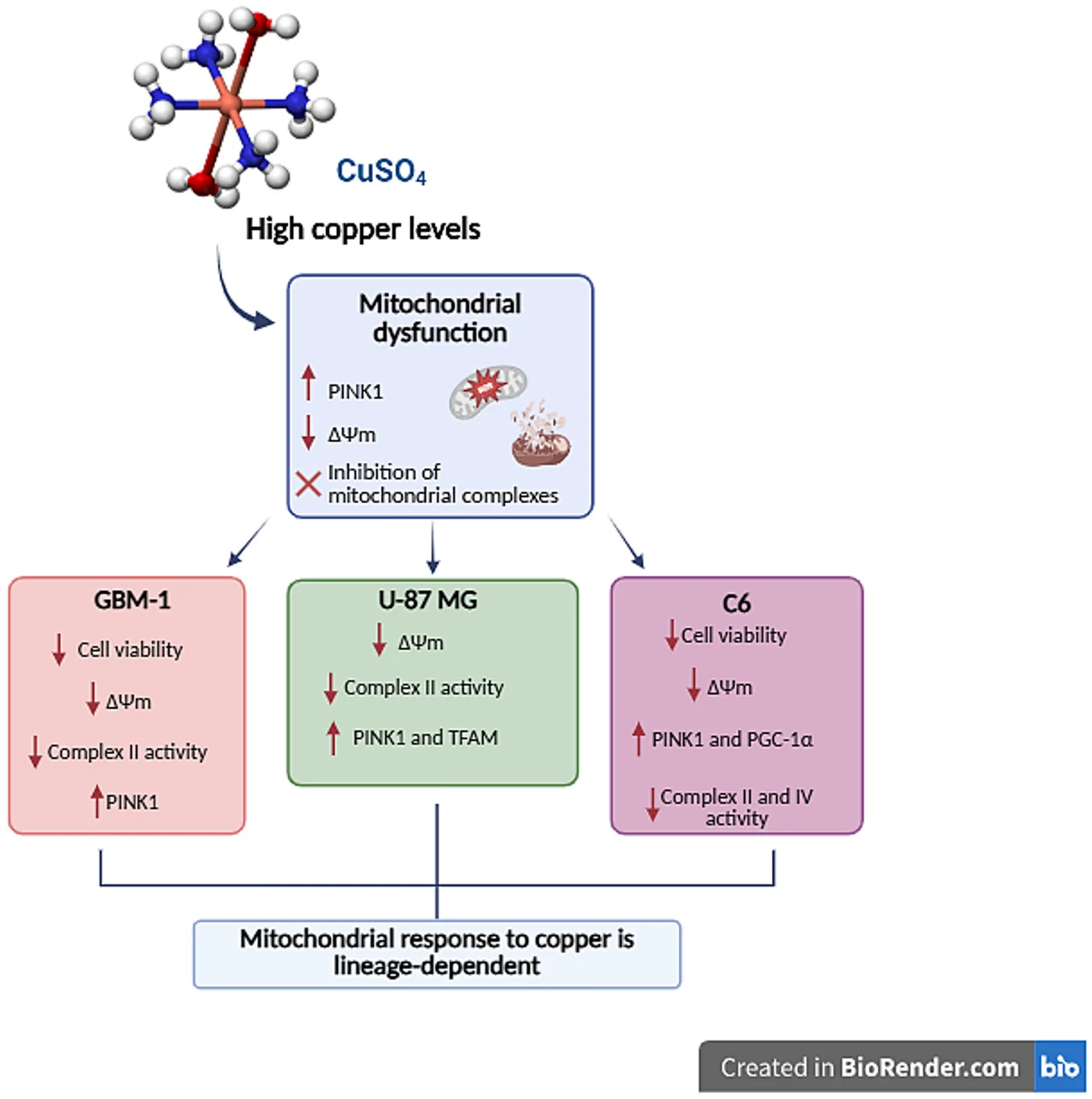

Exposure to high copper levels (CuSO_4_) promotes mitochondrial dysfunction in glioma cells, including loss of mitochondrial membrane potential (ΔΨm), and impairment of respiratory chain activity, besides increasing PINK1. Indeed, U-87 MG cells display increased TFAM expression, and C6 cells exhibit increased PGC-1α expression, consistent with activation of mitochondrial biogenesis pathways. Created in BioRender.com

## Introduction

Glioblastoma (GBM) is a highly aggressive and invasive malignant tumor, and the most common primary brain tumor worldwide (Miller et al. 2021; Sung et al. 2021). In the United States, 88,186 deaths were attributed to malignant brain and other CNS tumors between 2018 and 2022 (Price et al. 2025), while in Europe, an annual incidence rate of 4.8 cases per 100,000 inhabitants for astrocytic tumors is estimated (Crocetti et al. 2012). In Brazil, the incidence rate of central nervous system (CNS) tumors was 5.62 cases per 100,000 inhabitants among men and 5.17 among women during the 2018–2019 period, reflecting a similar global increase in diagnoses (Godoi et al. 2022; Marta et al. 2021; Santos et al. 2023).

Gliomas are classified into grades I-IV according to the World Health Organization (WHO) malignancy scale, with GBM being a grade IV tumor characterized by high malignancy and treatment resistance (Cimino et al. 2017). The standard treatment for GBM includes surgery, radiotherapy, and chemotherapy with temozolomide (Stupp et al. 2005). Still, the effectiveness of these approaches is limited by the functional heterogeneity of tumor cells, resulting in an average post-diagnosis survival of only 12–15 months (Villa et al. 2018).

Treatment resistance is a major challenge in GBM regression, driving interest in new therapies (Rong et al. 2022). In this context, the use of copper compounds for tumor treatment has been investigated due to their anticancer properties (Qu et al. 2025; Zhang B. et al. 2023). Copper nanoparticles, designed to transport and deliver copper to tumor cells, have been a target of studies investigating antitumoral activity (Naikoo et al. 2021). Elesclomol, a drug used to increase intracellular copper concentration, has also been studied in tumor cell lines, as has disulfiram, another copper ionophore that has been screened as an effective drug for cancer suppression (Xie J. et al. 2023; Zheng et al. 2022).

High copper levels are cytotoxic, primarily due to oxidative stress and protein inhibition, leading to tumor cell death (Letelier et al. 2005; Tang et al. 2022; Uriu-Adams and Keen 2005). Excess copper could induce cell death in different ways involving mitochondria, the main site of copper storage within cells (Dodani et al. 2011). Under aggressive tumor growth conditions, glioma cells increase mitochondrial energy production in contrast to glycolysis to supply the high biosynthetic and bioenergetic demands of GBM malignancy (Dong et al. 2023; Maher et al. 2012; Monzel et al. 2023). Therefore, mitochondria emerge as a promising target for copper-based therapies. In addition, a new form of copper-dependent cell death termed cuproptosis was described, characterized by the accumulation and aggregation of copper with lipoic acid-containing proteins in the tricarboxylic acid cycle. This results in lipoic acid protein aggregation and destabilization of mitochondrial iron-sulfur cluster proteins, causing acute proteotoxic stress and cell death (Tsvetkov et al. 2022; Zulkifli et al. 2023). Taken together, this body of evidence highlights the cytotoxic mechanisms triggered by elevated copper levels and reinforces the rationale for exploring this metal modulation as a therapeutic strategy for GBM.

In this context, the effects of high copper levels on different mitochondrial parameters in human GBM cell lines (GBM-1 and U-87 MG) and in a rat astroglioma cell line (C6) were assessed, mainly considering the heterogeneity of tumor cells. In this in vitro study, analyses will be conducted to investigate the effects of high copper levels on tumor cell energy metabolism and to determine whether therapeutic strategies aimed at increasing intracellular metal levels would be effective for treating GBM.

## Material and Methods

### Cell culture

The GBM-1 human glioblastoma cell line was obtained from the Celso Ramos Hospital in Florianópolis, Santa Catarina, Brazil, as described in Bittencourt and colleagues (Bittencourt et al. 2016), while U-87 MG human glioblastoma cells were obtained from Rio de Janeiro Cell Bank (BCRJ). C6 rat glioma cell line was obtained from the American Type Culture Collection. GBM-1 cells were cultured in flasks in Dulbecco’s Modified Eagle’s Medium/Nutrient mixture F-12 Ham (DMEM/F12), 1 U/mL penicillin, 1 μg/mL streptomycin, and 2.5 μg/mL amphotericin B, supplemented with 10% fetal bovine serum (FBS). U-87 MG and C6 cell lines were cultured in flasks in DMEM containing 1 U/mL penicillin, 1 μg/mL streptomycin, and 2.5 μg/mL amphotericin B, supplemented with 5% or 10% FBS (C6 and U-87 MG, respectively). The cells were maintained at 37 °C with at least 95% relative humidity and in a 5% CO_2_ atmosphere. After reaching confluence, the cells were trypsinized and cultured in separate plates. Copper sulfate (CuSO_4_) (Dinâmica, BR) exposure was performed by diluting it into culture medium with 1% FBS (300, 600, 900, and/or 1200 µM of CuSO_4_) for 24 h. Controls (CuSO_4_ 0 µM) were run in parallel, and only culture medium with 1% FBS was added to these wells. All the reagents used to maintain the cell culture were purchased from Sigma-Aldrich or from VitroCell (FBS).

### Cell viability

For the MTT and neutral red (NR) assays, cells were cultured in 96-well plates and, after reaching confluence, the cells were exposed to CuSO_4_ as described above. Post-treatment, cells were incubated with MTT (Sigma-Aldrich) at 37 °C, in the following concentrations and times, depending on the cell line: GBM-1 for 60 min (0.5 mg/mL), U-87 MG for 45 min (0.25 mg/mL), and C6 for 20 min (0.1 mg/mL). The produced formazan was solubilized with DMSO, and absorbance was measured at 540 nm using a plate reader (TECAN Infinite M200) (Mosmann 1983). For the NR assay, cells were incubated with NR solution (0.04 mg/mL, Sigma-Aldrich) for 4 h at 37 °C in a humidified atmosphere (95% O_2_ and 5% CO_2_). Following incubation, cells were washed and treated with a dye extraction solution (50:49:1 (v/v) 96% ethanol:distilled water:glacial acetic acid) for 10 min, and absorbance was measured at 540 nm using the plate reader (Repetto et al. 2008). Results were expressed as a percentage (%) of controls (considered as 100% of viability).

### Enzymatic activity

For evaluating mitochondrial respiratory chain enzyme activity, cells were cultured in 6-well plates at a density of 250,000 cells/mL. Post-treatment with CuSO_4_, cells were homogenized in phosphate buffer pH 7.4, containing 0.3 M sucrose, 5 mM MOPS, 1 mM EGTA, and 0.1% bovine serum albumin (BSA). Samples were transferred through a 1 mL syringe tip several times on ice and collected in a microtube. The homogenates were centrifuged at 1,000 × *g* for 5 min at 4 °C. The pellet was discarded, and the supernatants were used to measure the activity of mitochondrial respiratory chain complexes. The homogenate protein content was quantified using the Lowry method, using BSA as the standard (Lowry et al. 1951).

Complex I activity was assessed by measuring the reduction of potassium ferricyanide at 420 nm for 5 min, as described by Cassina and Radi (Cassina and Radi 1996). Complex II activity was measured by 2,6-dichloroindophenol reduction at 600 nm (Fischer et al. 1985), and complex IV activity by cytochrome c oxidation (Rustin et al. 1994), for 5 min. Enzyme activities were measured spectrophotometrically using a plate reader (Spectramax 190), and the data were expressed as nmol/min/mg of protein.

### Mitochondrial membrane potential (ΔΨm)

The fluorescent probe JC-1 (5,5’,6,6’-tetrachloro-1,1’,3,3’-tetraethylbenzimidazolylcarbocyanine iodide, Invitrogen) was used to analyze mitochondrial membrane potential (ΔΨm). Following exposure to various concentrations of CuSO_4_ and 0.5 mM rotenone (positive control) in 96-well plates, cells were incubated with JC-1 (5 μg/mL) for 25 min at 37 °C. Fluorescence was measured using the TECAN Infinite M200 plate reader with excitation at 485 nm and 535 nm, and emission at 530 nm and 590 nm. Results were expressed as the 590/530 fluorescence ratio and as a percentage of controls (100%).

### PINK1 immunocontent

Cells were grown in 12-well plates with coverslips and exposed to 600 µM CuSO_4_, given the decrease in cell viability observed in the GBM-1 and C6 cell lines. Moreover, an additional concentration of 900 µM CuSO_4_ was used in U-87 MG, since this line has demonstrated lower sensitivity to metal excess than the other glioma cell lines with respect to viability.

After 24 h of exposure to CuSO_4_, the cells were fixed with 4% paraformaldehyde (Sigma-Aldrich) for 1 h. Then, cells were permeabilized with 0.25% Triton X-100 in PBS (phosphate-buffered saline) (10 min) and incubated for 30 min with 5% PBS-BSA to block nonspecific binding. Coverslips were incubated in a humidified chamber overnight at 4 °C with PINK1 rabbit polyclonal antibody (PINK1: PTEN-induced kinase, 1:100 in PBS-BSA 1%, Invitrogen, catalog # PA1-16604). Furthermore, the samples were incubated with Alexa® 488 anti-rabbit secondary antibody (1:1,000 in PBS-BSA 1%; Invitrogen, catalog #A-11008) at 37 °C for 1 h, followed by DAPI staining (1:5,000; Sigma-Aldrich). Finally, coverslips were mounted in FluoroMount (Sigma-Aldrich) and were observed under a conventional fluorescence microscope (Olympus BX53) (Sosa et al. 2012). Samples were analyzed using the NIH ImageJ imaging software. The total pixel intensity was determined, and data were expressed as the mean of optical density/cell.

### PGC-1α and TFAM mRNA relative content

PGC-1α (peroxisome proliferator-activated receptor gamma coactivator 1-α) and TFAM (mitochondrial transcription factor A) mRNA relative content was assessed in U-87 MG and C6 cell lines, based on primer validation and DNA profiles in these models. Cells were cultured in 6-well plates at a density of 250,000 cells/mL and exposed to 0 (controls) or 600 µM of CuSO_4_, which produced a marked reduction in mitochondrial membrane potential in both cell lines. The total RNA was extracted using TRI® Reagent (Sigma-Aldrich), according to the manufacturer’s instructions. The concentration and purity of RNA were determined using a NanoVue Plus™ spectrophotometer. RNA samples with values of 260/280 and 260/230 > 1.8 were selected for cDNA synthesis.

cDNA was synthesized from RNA using the High-Capacity cDNA Reverse Transcription Kit (Thermo Fisher), following the manufacturer’s manual. Gene expression was quantified using PowerUp™ SYBR™ Green Master Mix (Applied Biosystems) in a Stratagene Mx 3005P real-time PCR system, in the presence of forward and reverse primers at the concentrations listed in Table 1 (primers from Sigma-Aldrich). The reaction was carried out with the following protocol: 50 °C for 2 min, 95 °C for 2 min, followed by 50 cycles of 95 °C for 15 s, 55 °C for 22 s, and 72 °C for 1 min. The dissociation curve was analyzed in a subsequent step, with 15 s at 95 °C, 60 °C for 1 min, and 15 s at 95 °C. Results were normalized to the housekeeping gene ATP5B, as described in Del Pozo et al. (Del Pozo et al. 2016). Relative mRNA expression levels were calculated by the 2^−ΔΔCT^ method (Livak and Schmittgen 2001) and expressed as relative mRNA content compared to the control group.

**Table 1:** Primer sequences used for RT-qPCR analysis

Primer sequence for *Rattus norvegicus* – C6 cells			
Gene	Primer (nM)	Sequence 5’—3’	Reference
PGC-1α	1200	F: TTGAAAAAGCTTGACTGGCGT R: CAGGGCAGCACACTCTATGT	NM_031347
TFAM	300	F: GGAATCAAGACTGTGCGTGC R: AGAAACTGCAATGGCTCTGC	NM_031326.2
ATP5B	600	F: GGCACCAATCAAAATTCCT R: AGCCTCAGCATGAATAGGAG	(Hvid et al. 2011)
Primer sequence for *Homo sapiens sapiens –* U-87 MG cells			
PGC-1α	1200	F: TCAGAGGTAACAGCCTCCAG R: GGCTACAGCTGTGAGAGTCC	NM_001330753.1
TFAM	3000	F: CGGGTTCCAGTTGTGATTGC R: CAGCTTTTCCTGCGGTGAATC	NM_003201.3
ATP5B	600	F: GTGGGCTATCAGCCTACCCT R: CAAGTCATCAGCAGGCACAT	(Del Pozo et al. 2016)

### Statistical analysis

The results were presented as mean ± standard deviation (SD). The Shapiro–Wilk test was used to assess the normality of the data. Data with normal distribution were analyzed using analysis of variance (ANOVA), followed by Dunnett’s post hoc test. PINK1 analysis in U-87 MG showed no parametric distribution, so it was analyzed using the Kruskal–Wallis test followed by Dunn’s post hoc test. Student’s t-test (parametric data) or Mann–Whitney U test (non-parametric data, i.e., PINK1 measurement in GBM-1 and C6 cell lines, and gene expression in C6 cells) was used to compare only two groups. Data were considered significant when p ≤ 0.05. Analyses and graphs were performed using GraphPad Prism 9.4®.

## Results

### Cell viability

**Fig. 1:**
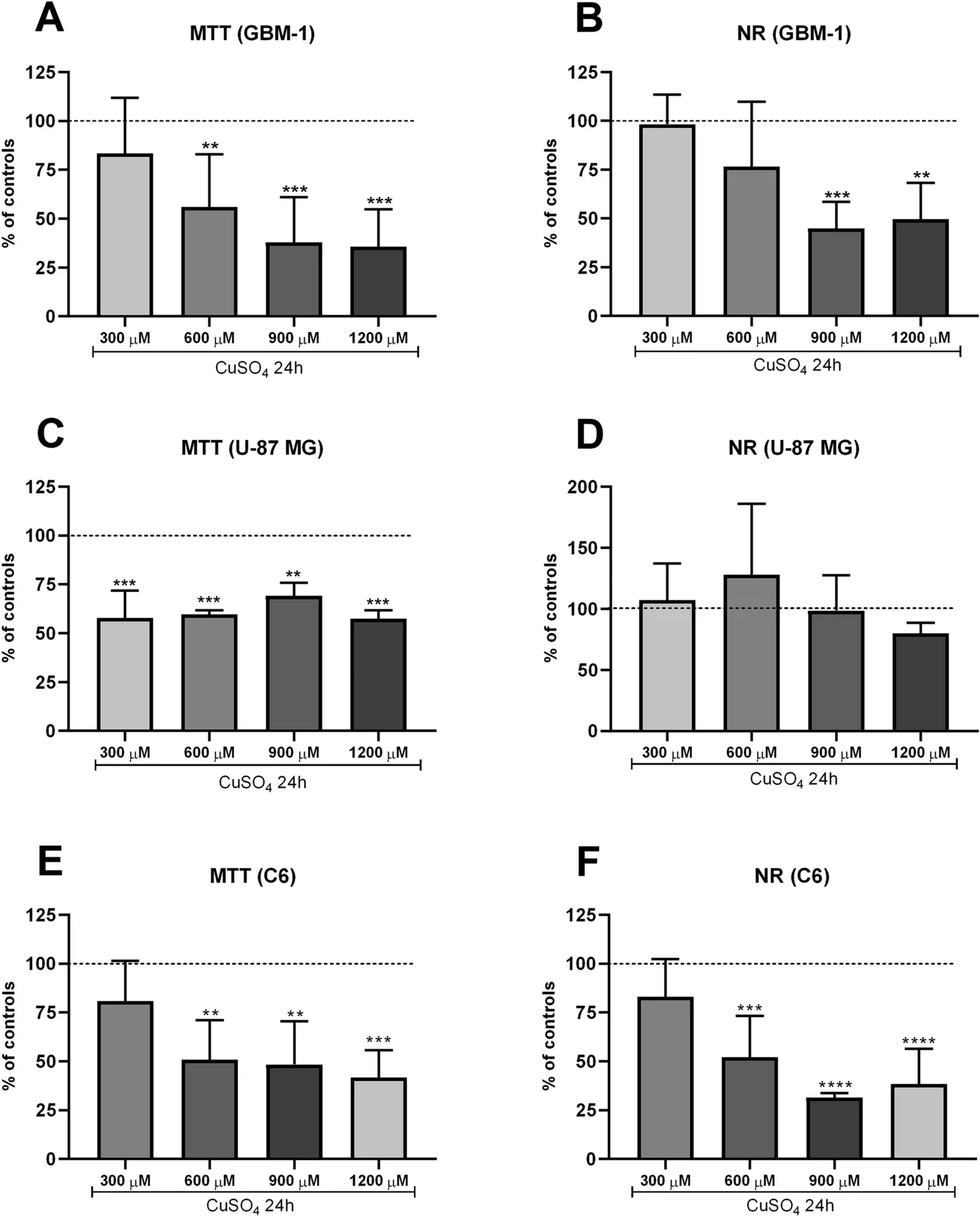
Viability of GBM-1, U-87 MG, and C6 cell lines assessed by MTT and neutral red (NR) assays, after 24 h of exposure to CuSO_4_ **A**, **C**, **E**: MTT reduction; **B**, **D**, **F**: neutral red dye absorption. The bars indicate the mean ± standard deviation (± SD) from 3 to 6 independent experiments (GBM-1: MTT N = 6, NR N = 5; U-87 MG: MTT N = 3, NR N = 3; C6: MTT N = 4, NR N = 5, with one outlier removed after analysis by the ROUT method [Q = 1%]), with each sample analyzed in duplicate. **(*p* < 0.01), ***(*p* < 0.001), and ****(*p* < 0.0001) compared to the control group. One-way ANOVA followed by Dunnett’s post hoc test

Cell viability assessed by the MTT reduction assay in the GBM-1 cell line decreased from 600 µM of CuSO_4_ after 24 h of exposure (Fig. 1A) [F_(4,25)_ = 9.885, p < 0.0001]. Additionally, cell viability was assessed using the NR assay, given that tetrazolium-based assays can be influenced by various redox-active chemicals, making the NR assay more precise and sensitive for evaluating the potential effects of copper compounds on cell viability (Gomez Perez et al. 2017). Using the NR assay in the GBM-1 cell line, viability decreased at 900 and 1200 µM of CuSO_4_ after 24 h of exposure (Fig. 1B) [F_(4,20)_ = 9.097, p < 0.001]. Regarding cell viability of the U-87 MG cell line exposed to CuSO_4_, this parameter decreased from 300 µM of CuSO_4_ after 24 h of exposure using the MTT method (Fig. 1C) [F_(4,10)_ = 18.53, p < 0.001]. Evaluating by the NR assay, viability did not decrease at any CuSO_4_ concentration after 24 h of exposure (Fig. 1D) [F_(4,10)_ = 0.8790, p = 0.5101]. Regarding C6 cells viability, it declines from 600 µM of CuSO_4_ when both methods were used (Fig. 1E, F) [MTT: F_(4,15)_ = 8.179, p < 0.01; NR: F_(4,19)_ = 17.03, p < 0.0001].

### Mitochondrial physiology

**Fig. 2:**
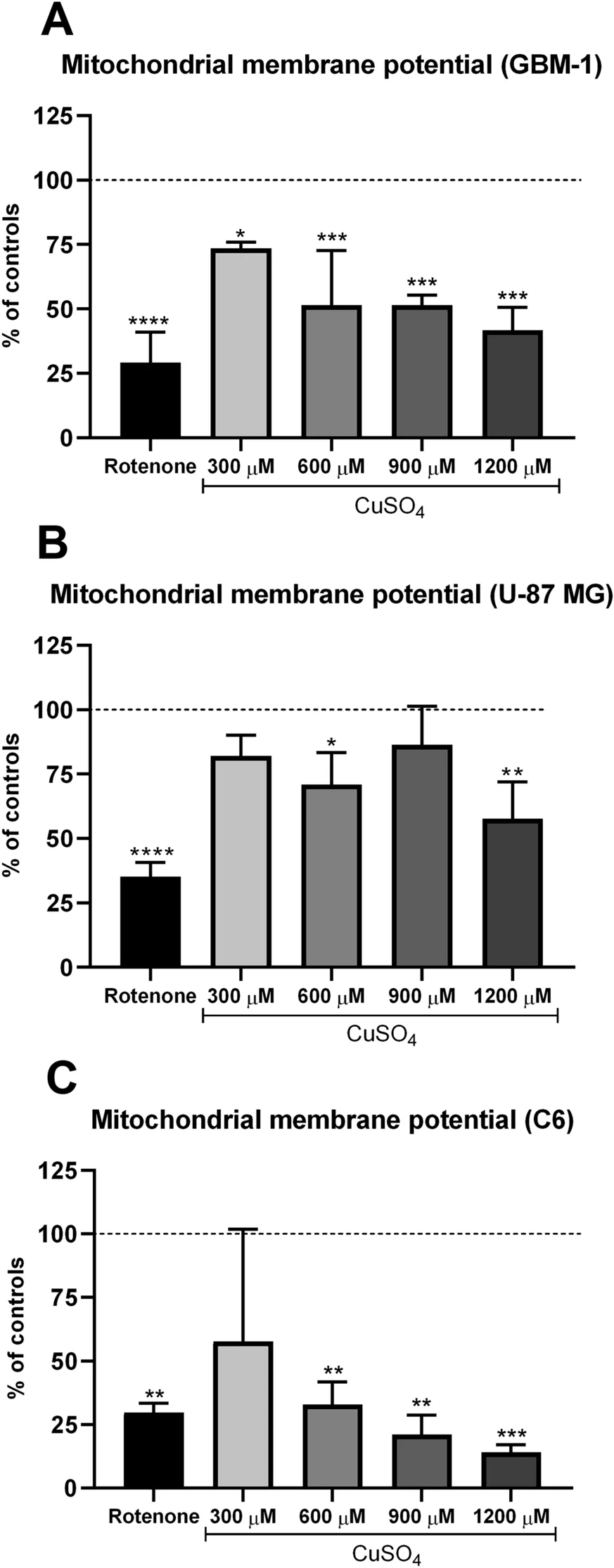
Mitochondrial membrane potential of GBM-1 (**A**), U-87 MG (**B**), and C6 (**C**) cell lines assessed by JC-1 probe after 24 h of exposure to CuSO_4._ Rotenone (0.5 mM) was used as a positive control. The bars indicate the mean ± standard deviation (± SD) from 3 independent experiments (N = 3), with each sample analyzed in triplicate. *(*p* < 0.05), **(*p* < 0.01), ***(*p* < 0.001), and ****(*p* < 0.0001) compared to the control group. One-way ANOVA followed by Dunnett’s post hoc test

ΔΨm was reduced at all tested concentrations in the GBM-1 cell line. In U-87 cells, a decrease was observed at 600 and 1200 µM CuSO_4_, whereas in C6 cells the reduction occurred from 600 µM onward (Fig. 2) [GBM-1: F_(5,12)_ = 16.68, p < 0.0001; U-87 MG: F_(5,12)_ = 14.12, p < 0.001; C6: F_(5,12)_ = 8.636, p < 0.01]. In this essay, rotenone (0.5 mM) was used as a positive control because it inhibits complex I activity, thereby reducing ΔΨm.

**Fig. 3:**
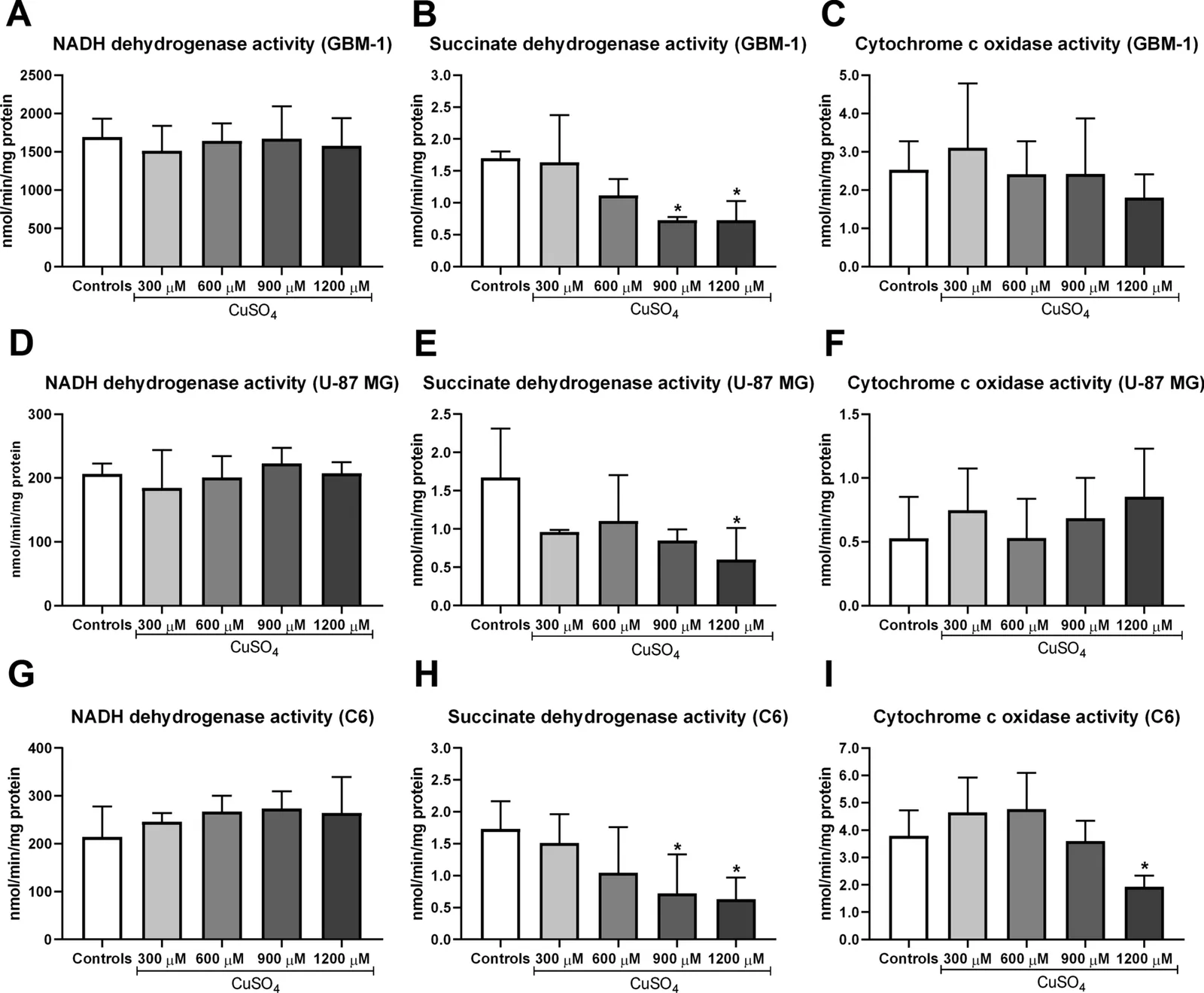
Enzymatic activity of mitochondrial complexes from GBM-1, U-87 MG, and C6 cell lines was assessed by NADH dehydrogenase, succinate dehydrogenase, and/or cytochrome c oxidase after 24 h of exposure to CuSO_4_. The bars indicate the mean ± standard deviation (± SD) from 3 to 5 independent experiments (GBM-1: Complex I N = 4, Complex II N = 3, Complex IV N = 4; U-87 MG: Complex I N = 3, Complex II N = 3, Complex IV N = 5; C6: Complex I N = 4, Complex II N = 5, Complex IV N = 5), with each sample analyzed in duplicate. *(*p* < 0.05) compared to the control group. One-way ANOVA followed by Dunnett’s post hoc test

Additionally, the mitochondrial function was assessed by measuring the enzymatic activities of NADH dehydrogenase (Complex I), succinate dehydrogenase (Complex II), and cytochrome c oxidase (Complex IV). NADH dehydrogenase activity after 24 h of CuSO_4_ exposure did not show any change at any copper concentrations (Fig. 3A, D, G) [GBM-1: F_(4,15)_ = 0.2089, p = 0.9299; U-87 MG: F_(4,10)_ = 0.4898, p = 0.7436; C6: F_(4,15)_ = 0.9273, p = 0.4742]. However, the succinate dehydrogenase activity was reduced at the higher CuSO_4_ concentrations in the three cell lines (Fig. 3B, E, H) [GBM: F_(4,10)_ = 4.595, p < 0.05; U-87 MG: F_(4,14)_ = 2.477, p < 0.05; C6: F_(4,20)_ = 4.177, p < 0.05]. Cytochrome c oxidase activity decreased only in C6 cells exposed to 1200 µM of CuSO_4_ (Fig. 3C, F, I) [GBM-1: F_(4,15)_ = 0.6537, p = 0.6332; U-87 MG: F_(4,20)_ = 0.9042, p = 0.4802; C6: F_(4,20)_ = 6.479, p < 0.05].

### PINK1 immunocontent

**Fig. 4:**
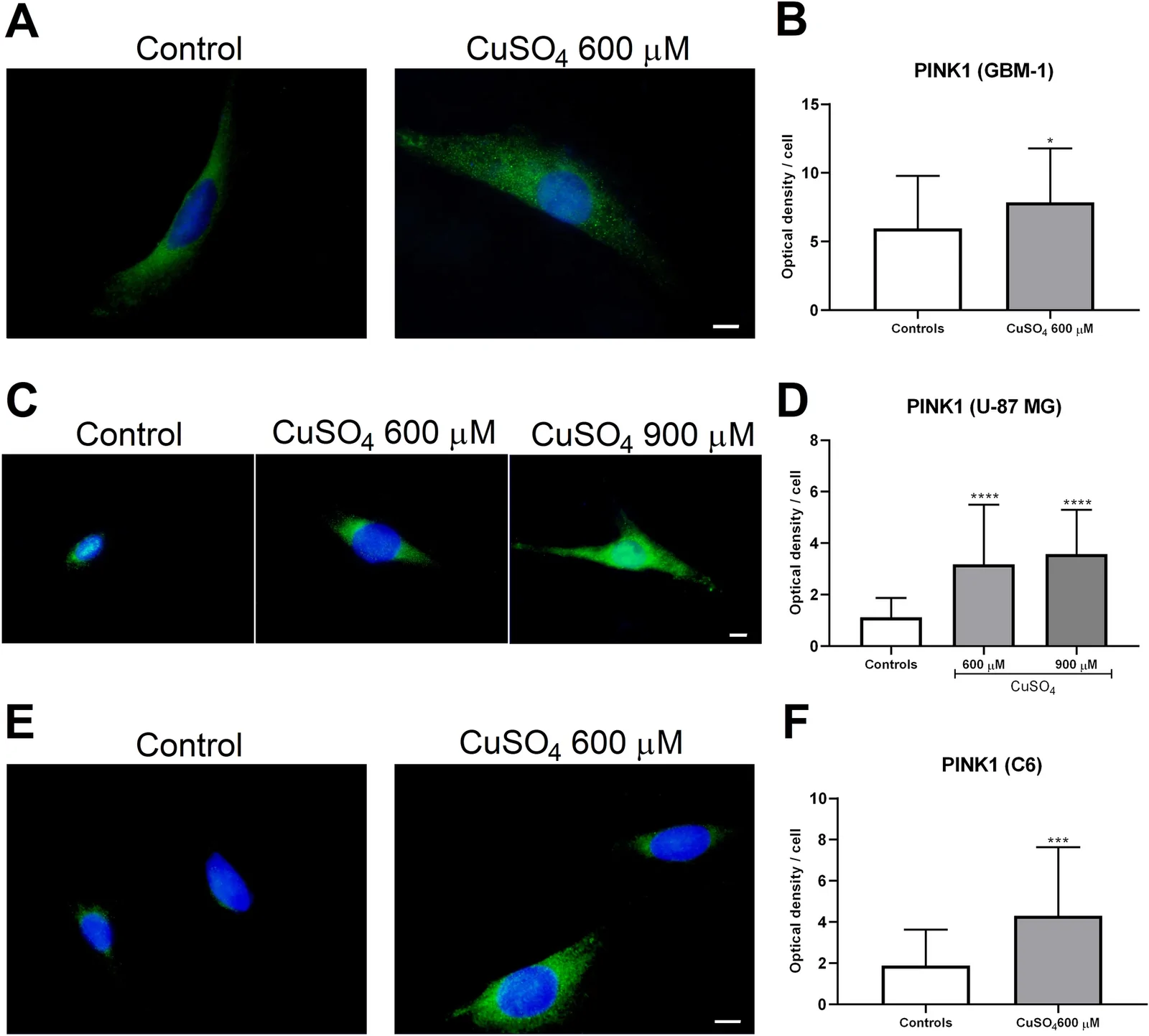
PINK1 immunostaining after 24 h of CuSO_4_ exposure in GBM-1 (**A**, **B**), U-87 MG (**C**, **D**), and C6 (**E**, **F**) cell lines. The bars indicate the mean ± standard deviation (± SD). 6–15 random photographs were taken for each sample of 3 independent experiments (GBM-1: N = 33 images for controls and N = 39 images for the group exposed to CuSO_4_ 600 µM; U-87 MG: N = 42 images for controls, N = 39 images for the group exposed to CuSO_4_ 600 µM, and N = 37 images for the group exposed to CuSO_4_ 900 µM. Four outliers were removed after analysis by the ROUT method [Q = 1%]). C6: N = 43 images for controls and N = 34 images for the group exposed to CuSO_4_ 600 µM. One outlier was removed after analysis by the ROUT method [Q = 1%]). *(*p* < 0.05); *** (*p* < 0.001); *** (*p* < 0.0001) compared to the control group. B, F: Mann–Whitney U test; D: Kruskal–Wallis followed by Dunn’s post hoc test. Scale bars: 5 µm

Figure 4 shows that the exposure of GBM-1, U-87 MG, and C6 cells to CuSO_4_ triggered an increase in PINK1 immunostaining [GBM-1: Mann–Whitney U_(70)_ = 456, p < 0.05; U-87 MG: Kruskal–Wallis_(2, 111)_ = 45.01, p < 0.0001; C6: Mann–Whitney U_(74)_ = 364, p < 0.001].

### PGC-1α and TFAM gene expression

**Fig. 5:**
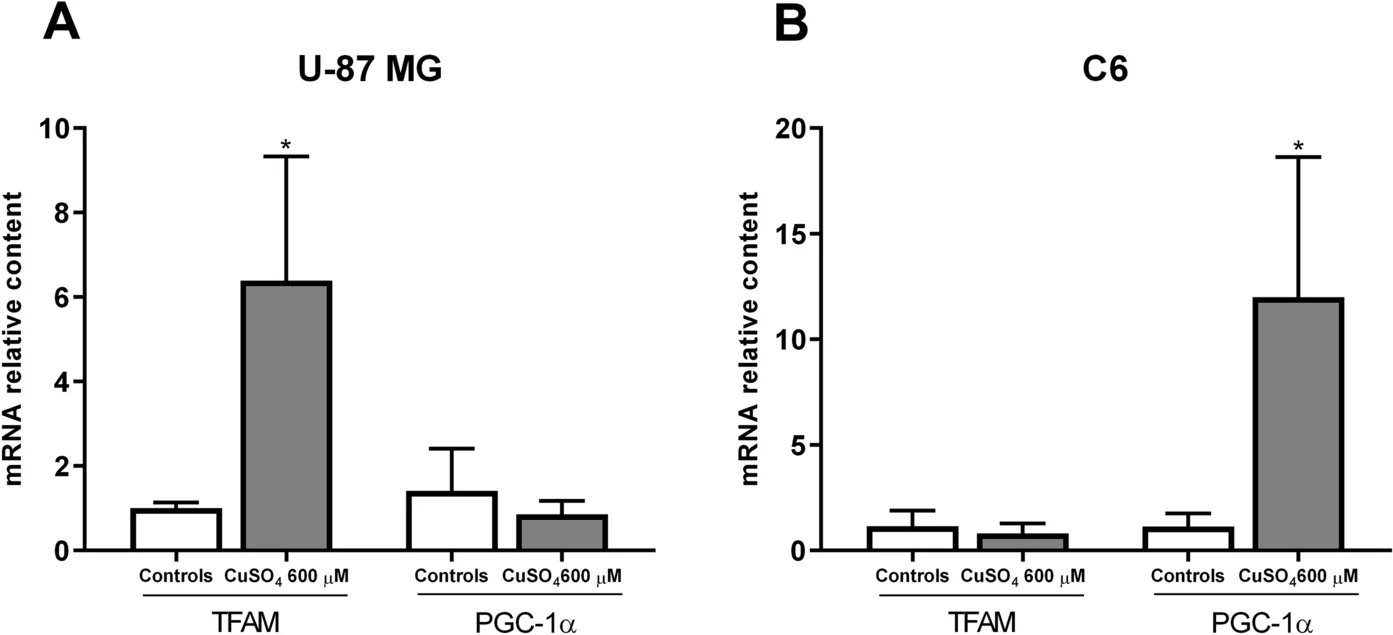
TFAM and PGC-1α mRNA relative content of U-87 MG (**A**), and C6 (**B**) cell lines after 24 h of exposure to CuSO_4._ The bars indicate the mean ± standard deviation (± SD) from 4 independent experiments (N = 4), with each sample analyzed in triplicate. *(*p* < 0.05) compared to the control group. A: Student’s t-test, B: Mann–Whitney U test

Considering that C6 appears to be the most susceptible cell line to CuSO_4_ deleterious effects and the U-87 MG cell line the lowest (considering the cell viability evaluated by NR assay), PGC-1α and TFAM mRNA levels were analyzed in these lines. In the U-87 MG cell line, an increase in the TFAM mRNA levels was observed after 24 h of treatment with 600 µM CuSO_4_ [TFAM: t_(6)_ = 3.657, p < 0.05; PGC-1α: t_(6)_ = 1.052, p = 0.3331] (Fig. 5A), while in the C6 cells, there was an increase in the PGC-1α relative mRNA content [TFAM: Mann–Whitney U_(6)_ = 4, p = 0.3429; PGC-1α: Mann–Whitney U_(6)_ = 0, p < 0.05] (Fig. 5B).

## Discussion

The growing interest in developing new therapies for GBM includes the use of copper compounds, given their anticancer properties. Copper ionophores have been studied to increase intracellular copper concentration because they bind copper in the Cu^2+^ form, transport it to the mitochondria, where it is reduced to Cu^+^, and induce cell death (Buccarelli et al. 2021; Jiao et al. 2025; Zheng et al. 2022; Zulkifli et al. 2023). These compounds aim to modify bioenergetic function, potentially uncoupling oxidative phosphorylation and inhibiting electron transport activity, promoting copper-mediated cytotoxicity (Hasinoff et al. 2014; Ma et al. 2019; Modica-Napolitano et al. 2019; Shimada et al. 2018). High copper levels could induce tumorigenic cell death through multiple pathways, including apoptosis, ferroptosis, and cuproptosis, all of which involve mitochondria.

In this way, mitochondria are a potential target for GBM treatment because they are involved in respiration and energy production, which are essential for the growth and development of the GBM tumor phenotype (Barthel et al. 2022). Excess copper disrupts redox homeostasis and oxidative phosphorylation, critical processes in the bioenergetics of cancer cells (Wu et al. 2023). In this context, analyzing the effects of high copper levels exposure on various aspects of the mitochondrial life cycle in human GBM and rat glioma cells helps us understand how copper-mediated induction of mitochondrial stress can be exploited as a therapeutic strategy for glioma treatment. Moreover, it is important to highlight that GBM-1 is a primary lineage long established as highly drug-resistant, and that the DNA profile of the U-87 MG glioma cell line, which is publicly available and widely used, is quite different from that of the original cell line (Allen et al. 2016). In addition, the C6 rat glial tumor cell line is the most commonly used model of a rodent brain tumor and is widely used to observe the efficacy of antitumoral therapies (Sahu et al. 2022). So, the use of different cell lines makes it valuable for comparing glioma lineages and obtaining more reliable results.

Herein, two methods were used to assess cell viability, as alternative methods to MTT have already been shown to be necessary when evaluating cells treated with copper compounds (Gomez Perez et al. 2017). For example, the U-87 MG cell line results indicate potential biases in cell viability analysis across different methods. In the MTT technique, for instance, copper may interact with the tetrazolium compound, which could explain the reduction in cell viability observed with this method, whereas no such change was observed with the NR method. In fact, there is a lack of literature comparing these two methods in glioma cell lines, although some studies suggest that comparing methods for analyzing cell viability in copper-treated cells increases the reliability and accuracy of the results (Chiba et al. 1998; Fotakis and Timbrell 2006; Gomez Perez et al. 2017). The viability data presented here are consistent with those previously demonstrated in other human and animal glioma lineages (Li et al. 2013; Shaligram and Campbell 2013; Stoeberl et al. 2025; Witt et al. 2021).

Moreover, the MTT reduction is dependent on mitochondrial dehydrogenases activity (Mosmann 1983), which could reflect the mitochondrial dysfunction observed after CuSO_4_ exposure. Thus, the MTT reduction used here to verify the cell viability could be influenced by that. In this way, in GBM-1, U-87 MG, and C6 cells, ΔΨm and succinate dehydrogenase activity were significantly reduced after 24 h of CuSO_4_ exposure at higher copper concentrations. The ΔΨm, crucial for ATP production, is primarily generated by protons pumping into the intermembrane space through the respiratory chain complexes. Complex I (NADH dehydrogenase) plays a significant role in this process by pumping four protons from a molecule of NADH into the intermembrane space. Although Complex II (succinate dehydrogenase) does not pump protons directly, it is essential for activating Complexes III and IV (cytochrome c oxidase), which together pump six more protons (four by Complex III and two by Complex IV), thereby establishing the membrane potential necessary for ATP synthesis (Nelson and Cox 2012). Thus, the decrease in the succinate dehydrogenase activity could be related to the changes in ΔΨm, since this complex donates electrons to Complex III and IV, although it does not directly pump H^+^ to the intermembrane space. Indeed, in C6 cells (a more sensitive cell lineage to high copper levels), Complex IV activity was also impaired, further contributing to the reduction in ΔΨm. In human cell lineages, the absence of significant changes in Complex IV activity suggests that Complexes I and III could effectively compensate for electron transport, partially maintaining the functionality of the respiratory chain (Lapuente-Brun et al. 2013; Maranzana et al. 2013; Milenkovic et al. 2017; Protasoni et al. 2020).

Impairment of mitochondrial function due to high copper levels could induce mitochondrial-selective autophagy to limit excessive ROS generation. In this context, mitophagy is a mitochondrial quality-control process that targets damaged mitochondria by forming autophagosomes, which then fuse with lysosomes for degradation and removal (Onishi et al. 2021). The classical mitophagy pathway involves PINK1 and Parkin; in healthy mitochondria, PINK1 is transported to the inner mitochondrial membrane and subsequently degraded (Liu L. et al. 2023; Onishi et al. 2021). However, in damaged mitochondria with membrane depolarization, PINK1 translocation is inhibited, resulting in its accumulation on the outer mitochondrial membrane, promoting the initiation of the mitophagy pathway (Aschner et al. 2024; Onishi et al. 2021). In fact, high copper levels increase PINK1 immunoreactivity in the three glioma cell lines used in this study, a finding associated with the mitochondrial dysfunction cited above. Under these conditions, mitophagy could be stimulated as a protective mechanism to remove dysfunctional mitochondria (Aschner et al. 2024), despite future research is necessary to evaluate this hypothesis in glioma cells, such as using autophagy markers to confirm mitophagy induction. Several studies have demonstrated that copper exposure, even for short periods or at moderate concentrations, can regulate mitophagy-related pathways and promote mitochondrial elimination in different models (Aschner et al. 2024; Bai et al. 2023; Yang et al. 2021; Zhu et al. 2023). Researchers have also demonstrated that an increase in PINK1 could be related to tumoral proliferation and resistance (Zhang R. et al. 2017), which could be linked to an adaptive response to avoid copper cytotoxicity. Furthermore, it has been proposed that PINK1 expression could influence antitumoral effects, and modulating its increase could be a strategy to chemoresistance (Fujii et al. 2025; Liu H. et al. 2024; Yamashita et al. 2017).

Additionally, mitochondrial impairment could induce the expression of proteins involved in the biogenesis of new mitochondria, thereby restoring bioenergetics in cells treated with high copper levels. Considering this, herein was observed the mRNA content of the transcriptional coactivator PGC-1α and TFAM, both involved in mitochondrial biogenesis. PGC-1α induces the expression of NRF1 and NRF2 (nuclear respiratory factor 1 and 2), stimulating transcription of nuclear genes involved in mitochondrial protein synthesis and inducing TFAM expression, which is involved in mitochondrial DNA (mtDNA) replication and mtDNA gene expression (Cardanho-Ramos and Morais 2021; Hardie 2004a, b; Popov 2020; Wang et al. 2023). In fact, this pathway could be upregulated in tumor cells due to high energy demands, and the increased expression of PGC-1α or TFAM in copper-treated glioma cells suggests that these are associated with the observed mitochondrial dysfunction, as an adaptive mechanism in which mitochondrial biogenesis is activated to compensate for mitochondrial damage, forming new mitochondria and preserving cellular energy function (Cho et al. 2017; Nguyen et al. 2020). Recent studies have demonstrated that modulating TFAM expression can directly influence tumor cell phenotypes and survival. In metabolic glioblastoma subtypes, TFAM loss has been associated with oxidative stress and divergent cellular behaviors, suggesting that TFAM regulates proliferation, redox balance, and other characteristics of glioma cells (Cavalcante et al. 2025). Yet studies in other cancer models have shown that reduced TFAM expression inhibits tumorigenesis and impairs cellular bioenergetics, thereby reducing proliferation and survival (Xie D. et al. 2016). Therefore, targeting the PGC-1α-TFAM pathway in conjunction with copper-induced mitochondrial stress could limit glioma cells’ ability to recover through mitochondrial biogenesis. This also could represent a strategy for future therapeutic studies.

Taken together, the data presented in this study demonstrate an important relationship between copper toxicity, mitochondrial damage, PINK1 increase, and the PGC-1α-TFAM pathway in different lineages of glioma, with these mechanisms being interconnected and essential for glioma tumor survival in high copper level conditions, although additional studies are needed to assess whether the mechanisms of copper overload described here occur in vivo in glioma tumoral cells. Additionally, it is still necessary to measure how much copper is internalized by the cells after exposure to copper compounds, thereby obtaining the intracellular concentration of the metal that could be effective to result in GBM cell death.

Furthermore, in recent years, some authors have studied how copper can be specifically delivered to glioma tumoral cells, with mitochondria as a well-established target in this context (Chen et al. 2024; Liu M. M. et al. 2025). Understanding the mechanisms underlying copper toxicity in different types of tumor cell lines could help open new horizons for the development of increasingly specific and effective therapies for tumor treatment. Moreover, cell lines show significant differences in their dose–response and treatment responses to copper, making it essential to investigate the mechanisms underlying the tumor characteristics observed to develop methods to overcome treatment resistance.

## Data Availability

Data will be made available on request.
